# DNA methylation in primary myelofibrosis is partly associated with driver mutations and distinct from other myeloid malignancies

**DOI:** 10.1186/s13148-025-01877-1

**Published:** 2025-05-03

**Authors:** Esra Dursun Torlak, Vithurithra Tharmapalan, Kim Kricheldorf, Joelle Schifflers, Madeline Caduc, Martin Zenke, Steffen Koschmieder, Wolfgang Wagner

**Affiliations:** 1 Institute for Stem Cell Biology, RWTH Aachen University Medical School, 52074 Aachen, Germany; 2 Helmholtz-Institute for Biomedical Engineering, RWTH Aachen University Medical School, 52074 Aachen, Germany; 3 Department of Hematology, Oncology, Hemostaseology and Stem Cell Transplantation, Medical Faculty of RWTH Aachen University, University Hospital Aachen, 52074 Aachen, Germany; 4 Center for Integrated Oncology, Aachen Bonn Cologne Düsseldorf (CIO ABCD), Aachen, Germany

**Keywords:** Primary myelofibrosis, DNA methylation, Myeloid malignancies, MPN, AML, JMML, MDS, Epigenetic, CpG

## Abstract

**Background:**

Primary myelofibrosis (PMF) is a clonal blood disorder characterized by mutually exclusive driver mutations in *JAK2*, *CALR*, or *MPL* genes. So far, it is largely unclear if the driver mutations have a specific impact on DNA methylation (DNAm) profiles and how epigenetic alterations in PMF are related to other myeloid malignancies.

**Results:**

When we compared DNAm profiles from PMF patients we found very similar epigenetic modifications in *JAK2* and *CALR* mutated cases, whereas *MPL* mutations displayed less pronounced and distinct patterns. Furthermore, induced pluripotent stem cell (iPSC) models with *JAK2* mutations indicated only a moderate association with PMF-related epigenetic changes, suggesting that these alterations may not be directly driven by the mutations themselves. Additionally, PMF-associated epigenetic changes showed minimal correlation with allele burden and seemed to be largely influenced by shifts in the cellular composition. PMF DNAm profiles compared with those from other myeloid malignancies—such as acute myeloid leukemia, juvenile myelomonocytic leukemia, and myelodysplastic syndrome—showed numerous overlapping changes, making it difficult to distinguish PMF based on individual CpGs. However, a PMF score created by combining five CpGs was able to discern PMF from other diseases.

**Conclusion:**

These findings demonstrate that PMF driver mutations do not directly evoke epigenetic changes. While PMF shares epigenetic alterations with other myeloid malignancies, DNA methylation patterns can distinguish between PMF and related diseases.

**Supplementary Information:**

The online version contains supplementary material available at 10.1186/s13148-025-01877-1.

## Background

Malignancies are marked not only by somatic mutations but also by epigenetic modifications, though the interplay between the two remains largely unexplored. Myeloproliferative neoplasms (MPNs) offer a unique opportunity to investigate whether specific mutations lead to distinct epigenetic changes, as they feature unique driver mutations in Janus kinase 2 (*JAK2*), particularly at the amino acid position 617 (valine to phenylalanine, V617F), calreticulin (*CALR*), and myeloproliferative leukemia protein (*MPL*) [1]. MPN are categorized into various subentities, such as essential thrombocythemia (ET), polycythemia vera (PV) and primary myelofibrosis (PMF), which harbor these mutations at different frequencies [2], with *JAK2* V617F mutations occurring in almost all PV cases but only approximately 50–60% ET and PMF cases, *CALR* mutations occurring in 25–35% of ET and PMF but not PV cases, and *MPL* mutations occurring in 5–8% of ET and PMF but not PV cases [3].

DNA methylation (DNAm) at CG dinucleotides (CpGs) is an epigenetic mechanism that modulates chromatin structure, transcription, and splicing [4]. Prior research already demonstrated aberrant DNAm in MPN [5]. The different entities of MPN were shown to have similar DNAm changes, which increase during progression and may play an important role in the pathogenesis and leukemic transformation [6]. Recently, it has been suggested that DNAm could serve as a biomarker for the fibrotic progression in PMF [7]. However, it is so far unclear whether distinct driver mutations are associated with specific epigenetic modifications. Understanding these connections could yield valuable insights into disease mechanisms and identify potential therapeutic targets.

Epigenetic aberrations are also implicated in other myeloid disorders, including acute myeloid leukemia (AML), myelodysplastic syndromes (MDS), and juvenile myelomonocytic leukemia (JMML) [8]. However, a comprehensive study that compares these myeloid malignancies with respect to their DNAm profiles is yet elusive. Moreover, many previous studies have not adequately addressed how changes in cellular composition of a given blood sample may affect aberrant DNAm patterns in malignancies [9].

In this study, we have therefore systematically compared the DNAm profiles associated with different driver mutations in PMF and subsequently assessed how these profiles differ from those of other myeloid malignancies.

## Methods

### Blood samples

Peripheral blood mononuclear cells (PBMCs) of 28 patients diagnosed with PMF, 2 patients diagnosed with ET and 10 healthy donors were used for the study. A detailed description of the sample cohort is shown in Supplemental Table S1. PBMCs were isolated from peripheral blood via Ficoll density gradient centrifugation and DNA was isolated with the QIAamp DNA Mini Kit (Qiagen, Hilden, Germany).

### Analysis of mutational burden

We employed a clinically validated amplicon-based next-generation sequencing (NGS) panel (Truseq Custom Amplicon Kit, Illumina, San Diego, USA) to analyze the coding regions of 32 genes commonly associated with hematologic malignancies [10]. Variants were manually reviewed, applying a bidirectional frequency cutoff of > 1% for driver mutations and > 5% for additional mutations.

### Analysis of DNA methylation profiles

Genomic DNA of the above-mentioned samples was subjected to bisulfite conversion and analyzed using the Illumina human EPIC methylation microarray version 2 (EPIC v2; GSE277841). We also examined DNAm profiles of induced pluripotent stem cell (iPSC) lines of three PV donors that are either WT, homozygous or heterozygous for the *JAK2* V617F mutation (supplemental methods). iPSC clones and their iPSC-derived hematopoietic progenitors (iHPCs) were analyzed with EPIC version 1 (EPIC v1; GSE277890). Additionally, we included DNAm profiles of an earlier study on 4 PV, 4 MF, and 4 healthy control samples hybridized on the 450 k BeadChip for our validation cohort (GSE277866). All Illumina BeadChip microarrays were analyzed at Life and Brain (Bonn, Germany). Further details on preprocessing and DNAm analysis are provided in the supplemental methods.

To investigate the granulocyte bias observed by the epigenetic deconvolution, we utilized a dataset containing triple negative MPN samples (GSE156546: *n* = 25) [27] and a dataset with different subsets of hematopoietic stem and progenitor cells (GSE63409: *n* = 30) [11]. To mitigate the impact of variations in cellular composition, we retrieved 289 DNAm profiles of sorted human hematopoietic cell types from the GEO database, including B cells (*n* = 60), CD4 T cells (*n* = 63), CD8 T cells (*n* = 56), granulocytes (*n *= 34), monocytes (*n* = 61), NK cells (*n* = 6), and dendritic cells (*n* = 9; Supplemental Table S2). Pairwise comparisons of mean DNAm values across all cell types were conducted, focusing on CpGs that did not exceed a beta value threshold of 0.1 in any of these comparisons (393,675 CpGs). For further analysis, we concentrated on CpGs that were also detected in all datasets of myeloid malignancies (216,532 CpGs).

For comparison with other myeloid malignancies, we used DNAm datasets of MDS (GSE221745: *n* = 5; GSE152710: *n* = 73), JMML (GSE237299: *n* = 41), and AML (GSE212937: *n* = 5; GSE62298: n = 68), as well as, healthy control datasets of either peripheral blood (GSE141682: *n* = 42; GSE221745: *n* = 5) or bone marrow (GSE221745: *n* = 7; GSE124413: *n* = 40; GSE152710: *n* = 10 samples). For validation, we used available datasets of PMF (GSE152519: *n* = 35; GSE118241: *n* = 22), secondary MF (GSE118241: *n* = 17), ET (GSE156546: *n* = 32), AML (GSE159907: *n* = 316), pediatric AML (GSE133986: *n* = 64) and healthy controls (GSE118241: *n* = 6; GSE156546: *n* = 2; Supplemental Table S3).

### Additional methods

Methods for preprocessing and further analysis of DNAm profiles, correlation with gene expression data, generation and characterization of hematopoietic differentiation of iPSC lines, targeted bisulfite amplicon sequencing, and colony-forming unit (CFU) assays are detailed in the supplemental methods.

## Results

### Aberrant DNA methylation in primary myelofibrosis

**Fig. 1:**
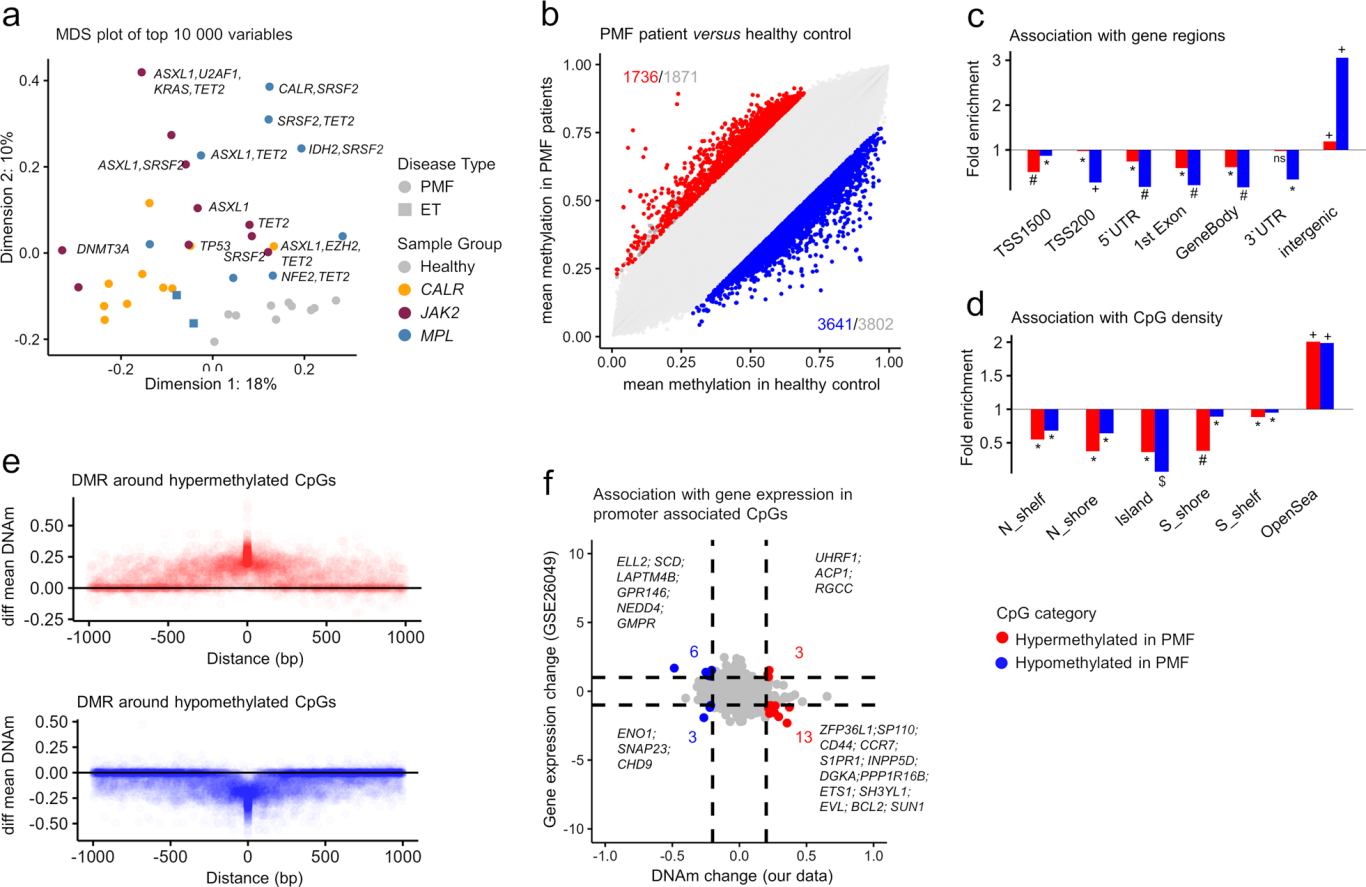
Aberrant DNA methylation in primary myelofibrosis. **a** Multidimensional scaling plot of DNA methylation profiles in PMF patients with different driver mutations (*JAK2*, *CALR*, *MPL*) and healthy controls (812,274 CpGs). **b** Scatter plot of mean methylation beta values of PMF patients and healthy controls. Significant hypo- and hypermethylated CpGs are indicated in blue and red (mean DNAm difference > 20%; adjusted *p*-values < 0.05). Gray numbers indicate all CpGs exceeding the mean DNAm difference > 20%, irrespective of statistical significance. **c**, **d**) Enrichment analysis of significant hyper- and hypomethylated CpGs in PMF patients in c genomic regions and **d**) CpG islands (Hypergeometric distribution: * = *p* < 0.05, # = *p* < 10^–10^, + = *p* < 10^–20^, and $ = *p* < 10^–100^. **e** Differential mean DNAm of CpGs adjacent to all significant hypermethylated and hypomethylated CpGs (1 kb window). **f** Comparison of DNA methylation and gene expression changes (GSE26049) between PMF patients and healthy controls, with genes showing significant differences in both categories highlighted

We examined DNAm profiles of peripheral blood mononuclear cells of PMF patients with *JAK2* V617F mutation (*n* = 10), *CALR* mutation (*n* = 10), *MPL* mutation (*n* = 10, including two ET samples), alongside healthy controls (*n* = 10; Supplemental Table S1). Multidimensional scaling plots (MDS plots) clearly separated DNAm profiles of PMF and healthy samples, whereas PMF samples with different driver mutations revealed some differences but were less clearly separated (Fig. 1a). While samples with additional mutations tended to cluster further away from controls, they did not exhibit a clear separation of specific mutations.

Comparing PMF (*n* = 28) and control samples, we identified 1,736 hypermethylated and 3,641 hypomethylated CpGs in PMF (adj *p* < 0.05; difference of mean DNAm > 20%; Fig. 1b; Supplemental Figure S1a,b). The most significantly hypermethylated CpGs were frequently linked to genes involved in the pathogenesis of hematological diseases, including *RUNX1* [12], *BRD4* [13], *SRSF2* [14], *SETBP1* [15] and *TNFSF10* [16]. Overall, the genes associated with differentially methylated CpGs were enriched in the Gene Ontology (GO) categories for immune response (Supplemental Figure S1c) and were predominantly located in intergenic regions (Fig. 1c), rather than with CpG islands commonly associated with promoter regions (Fig. 1d).

To assess whether aberrant DNAm affects single CpG sites or broader differentially methylated regions (DMRs), we analyzed DNAm in the surrounding of the 1,736 hyper- and 3,641 hypomethylated CpGs. A significant gain or loss of DNAm was observed within a 500 bp window around these CpGs (Fig. 1e). We also compared DNAm changes to gene expression profiles from a publicly available dataset with nine PMF samples (GSE26049). While no clear overall association was found, some candidate genes exhibited concordant changes in DNAm and gene expression, such as hypermethylated and downregulated *ZFP36L1*, linked to myelofibrosis progression [17] and *INPP5D* known to be downregulated by *JAK2* V617F [18]. Conversely, hypomethylated and upregulated genes included *LAPTM4B* [19], and *NEDD4* [20], both associated with oncogenic potential (Fig. 1f). To further validate these findings, we compared gene expression profiles of CD34 + sorted cells from PMF patients and controls (GSE53482) and found a similar association (Supplemental Figure S1d).

### Similar epigenetic effects from *JAK2* and *CALR* mutations

**Fig. 2:**
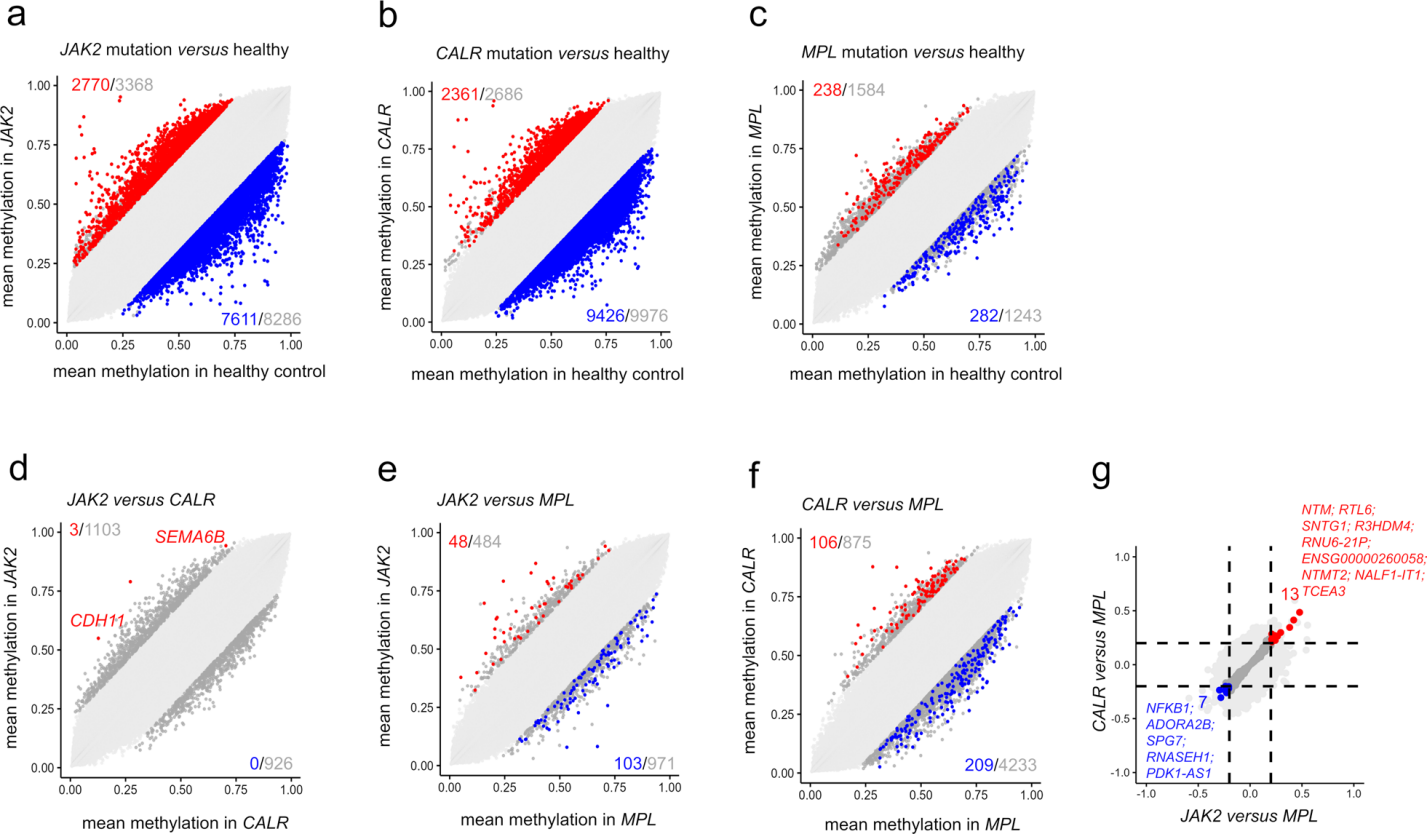
Changes in DNA methylation according to driver mutations. **a**-**c** Scatter plots illustrating the mean DNAm beta values in PMF patients compared to healthy controls, stratified by driver mutation: a) *JAK2* mutation, b) *CALR* mutation, and c) *MPL* mutation. **d**-**f** Additional scatter plots comparing the mean DNAm beta values of PMF patients based on their driver mutation: d) *CALR versus JAK2;* e) *MPL versus JAK2*; and f) *MPL versus CALR*. Significant hypo- and hypermethylated CpGs are indicated in blue and red, respectively (mean DNAm difference > 20%; adjusted *p*-values < 0.05). **g** Comparison of significantly differentially methylated CpGs between *JAK2* and *MPL versus CALR* and *MPL*, with gene names for overlapping CpGs highlighted

Next, we categorized PMF samples based on their specific driver mutation. Comparing healthy samples (*n* = 10) with MPN samples harboring specific driver mutations (*n* = 10 each), we identified 2,770 hyper- and 7,611 hypomethylated CpGs for *JAK2*, 2,361 and 9,426 for *CALR,* and 238 and 282, respectively, for *MPL* mutations (all adj *p* < 0.05; difference of mean DNAm > 20%; Fig. 2a-c). Thus, *MPL* mutations revealed fewer modifications than those with *JAK2* and *CALR* mutations, and this was also observed when we excluded the two ET samples with *MPL* mutation (882 hyper- and 598 hypomethylated CpGs). Notably, a direct comparison between *JAK2* and *CALR* samples revealed only three significant CpGs, indicating minimal differences in their impact on DNAm profiles (Fig. 2d). Comparison of *JAK2 versus MPL* (Fig. 2e) and *CALR versus MPL* (Fig. 2f) revealed a greater number of significant CpGs (Supplemental Table S4), suggesting that *MPL* mutations have distinct epigenetic consequences compared to *JAK2* or *CALR* mutations (Fig. 2g).

### Patient specific DNAm patterns in a *JAK2* iPSC model

**Fig. 3:**
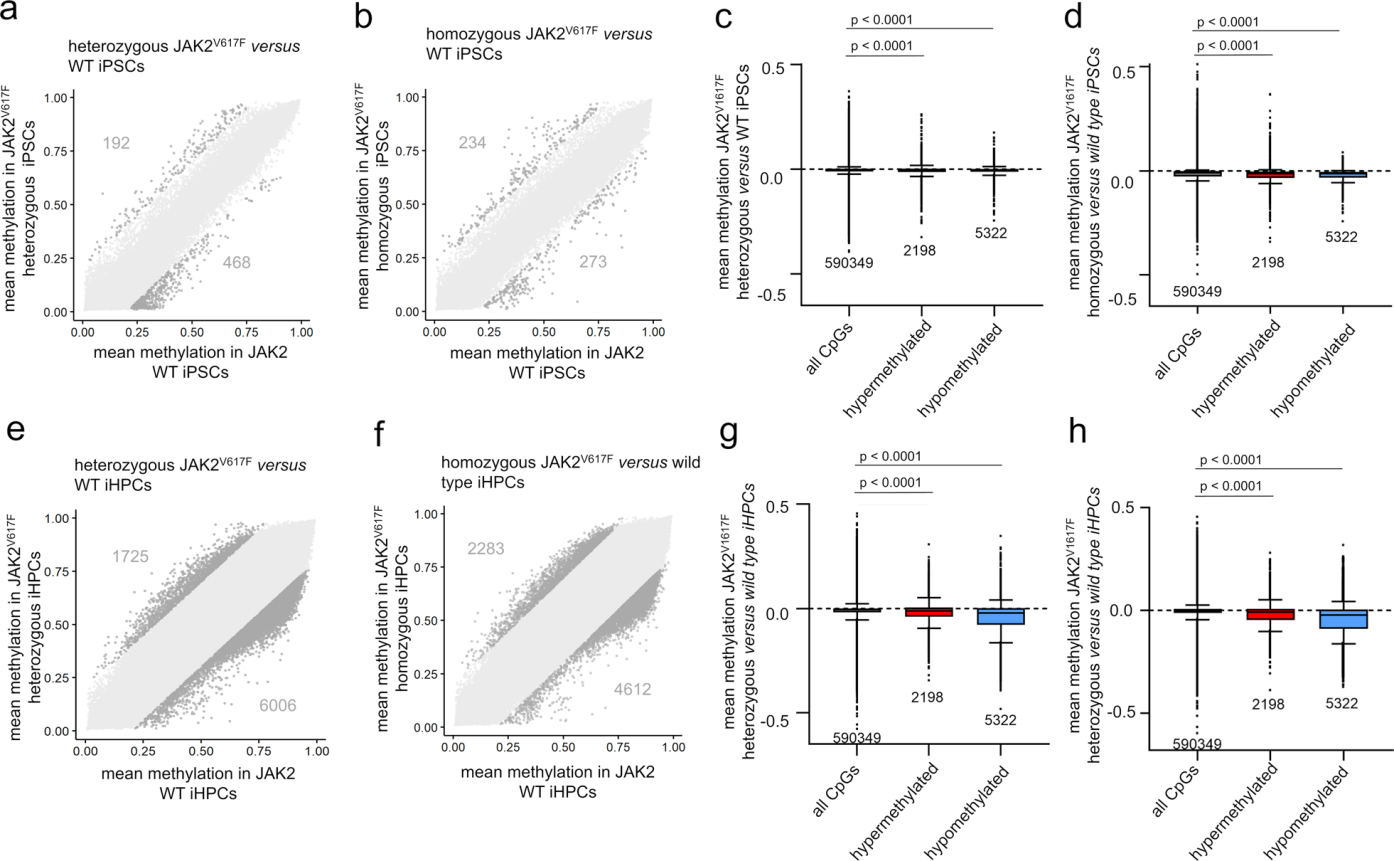
iPSCs with *JAK2* V617F fail to recapitulate disease-associated changes. **a**, **b** Scatter plots showing mean DNAm beta values of a) wild type (WT) *versus JAK2* V617F heterozygous (het) iPSCs, and b) WT *versus JAK2* V617F homozygous (hom) iPSCs. The numbers of CpGs with > 20% DNAm difference are indicated, but none reached statistical significance. **c**, **d** To determine if DNAm changes in PMF patients with *JAK2* V617F are reflected in iPSCs with or without *JAK2* V617F, we focused on CpGs that were significantly differentially methylated in *JAK2* V617F PMF *versus* healthy control (from Fig. 2a). Average DNAm changes were then analyzed in these CpGs in iPSCs with either c) WT *versus* heterozygous *JAK2* V617F, or d) WT *versus* homozygous *JAK2* V617F. **e**, **f** Following differentiation of iPSC lines into hematopoietic progenitor cells (iHPCs), scatter plots depict mean DNAm beta values for e) WT *versus JAK2* V617F heterozygous iHPCs, and f) WT *versus JAK2* V617F homozygous iHPCs (none of the CpGs reached statistical significance). Gray numbers indicate all CpGs exceeding the mean DNAm difference > 20%, irrespective of statistical significance. **g**, **h** The CpGs with significant differences in *JAK2* V617F PMF *versus* healthy controls were reanalyzed in iHPCs: g) heterozygous and h) homozygous *JAK2* V617F iHPCs exhibited an overall decrease in DNAm at CpGs that gained or lost methylation in *JAK2* V617F PMF. Statistical significance was evaluated using one-way ANOVA

To further explore the link between *JAK2* V617F mutations and epigenetic aberrations, we analyzed DNAm profiles in iPSC lines derived from three PV patients previously generated and independent of the above PMF samples, for each of them wild type (WT) *JAK2*, heterozygous (het), and homozygous (hom) *JAK2* V617F mutations [21, 22]. The iPSC model offers the advantage of working with clonal and homogenous cell populations, where all cells within a given cell population harbor either heterozygous or homozygous *JAK2* V617F mutations or no *JAK2* mutation. In the pluripotent state, no significant DNAm differences were observed between WT and either het or hom iPSC clones (adj *p* < 0.05; Fig. 3a, b). However, focusing on CpGs exhibiting significant hyper- or hypomethylation in the blood samples of *JAK2* V617F positive patients *versus* healthy controls (2,198 *versus* 5,322 CpGs; lower CpG numbers than in Fig. 2a due to the different EPIC array version), we noted a modest yet significant hypomethylation in *JAK2* V617F iPSCs compared to WT iPSCs (Fig. 3c, d; Supplemental Figure S2a, b).

To determine whether the impact of *JAK2* V617F mutations was masked in the pluripotent state, we differentiated these clones into hematopoietic progenitor cells (iHPCs; Supplemental Figure S3a). After 16 days, all clones generated non-adherent cells exhibiting typical hematological morphology, with flow cytometry confirming upregulation of various hematopoietic markers (Supplemental Figure S3b, c). Heterozygous and homozygous *JAK2* V617F iHPCs showed a bias toward CD235a/glycophorin A positive erythroid cells in line with our previous studies [21, 22]. DNAm profiles validated that iHPCs had exited the pluripotent state and were aligned toward mesodermal lineage (Supplemental Figure S3d, e) [23]. When comparing iHPCs to iPSCs, we identified 3,201 hypermethylated and 25,507 hypomethylated CpGs (adj *p* < 0.05; difference of mean DNAm > 20%; Supplemental Figure S3f). These CpGs were enriched in genes related to Gene Ontology categories including blood vessel development, regulation of signaling and migration, indicating that the differentiation captured epigenetic changes of hematopoietic development (Supplemental Figure S3g). However, even in iHPCs there were no significant differences between WT and *JAK2* V617F mutated clones. This lack of distinction might stem from inherent variability during the differentiation process (Fig. 3e, f).

Focusing on the CpGs with notable DNAm changes in *JAK2* V617F patients, we found that hypomethylated CpGs in *JAK2* V617F PMF also exhibited hypomethylation in iHPCs carrying the *JAK2* V617F mutation. However, also the hypermethylated CpGs in patients showed moderate hypomethylation in iHPCs (Fig. 3g, h). A direct comparison of DNAm changes associated with *JAK2* V617F in iHPCs and PMF patients revealed a moderate but significant association between hypomethylated regions (Supplemental Figure S2c, d). This could be linked to a partial recapitulation of MPN phenotype observed in the *JAK2* V617F iPSC clones. Overall, our findings suggest that the iPSC model does not accurately reflect the DNA methylation changes seen in PMF, indicating that the epigenetic alterations may not be directly driven by the *JAK2* V617F mutation alone.

### Epigenetic age is accelerated in primary myelofibrosis

**Fig. 4:**
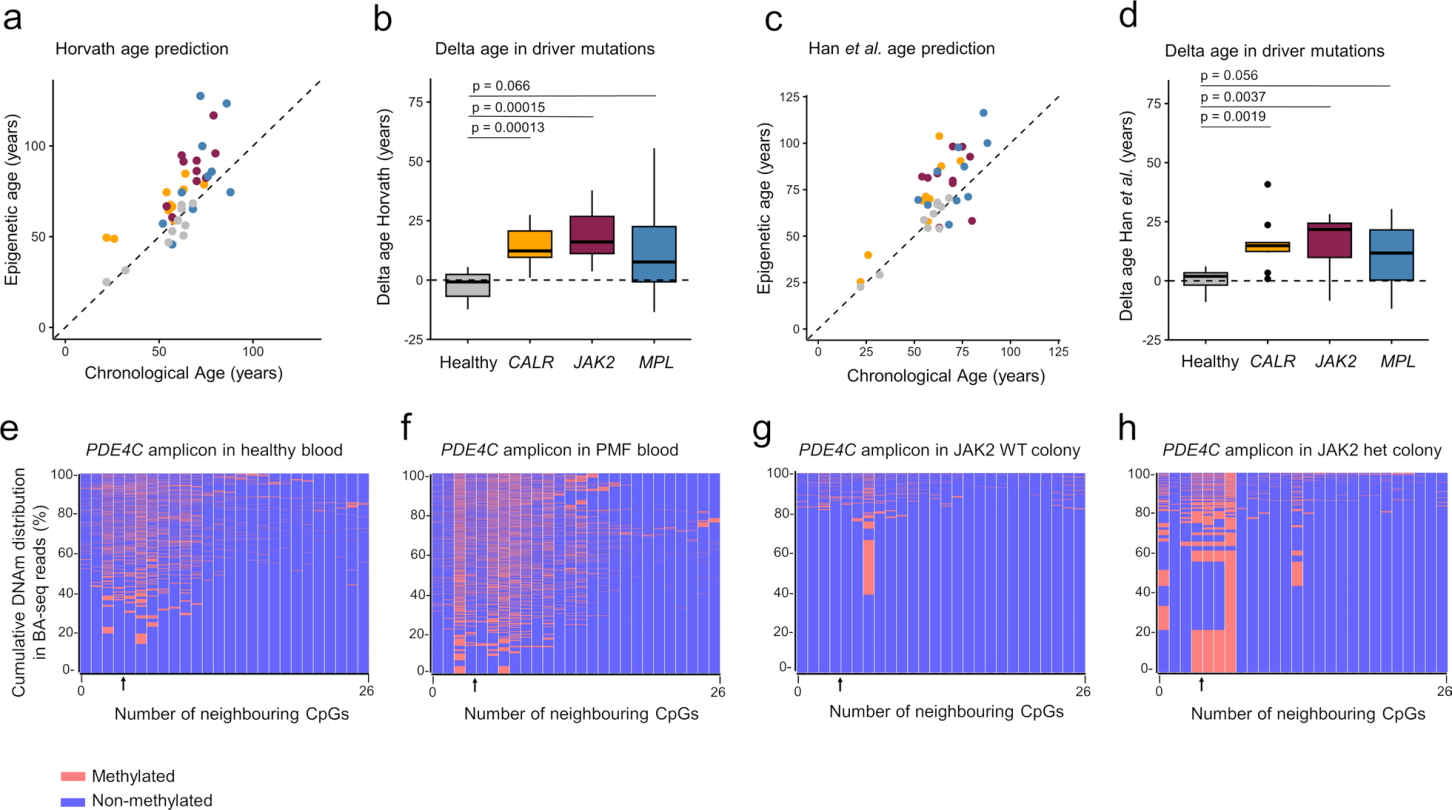
Age-associated DNAm changes in primary myelofibrosis. **a**–**d** The correlation between epigenetic age predictions with chronological age and the difference between predicted and chronological age (delta-age) was determined with epigenetic clocks developed by a,b) Horvath [26] and c,d) Han et al. [25]. Statistical significance was determined using an unpaired *t*-test. **e**–**h** The DNAm at an age-associated region in *PDE4C* was analyzed by bisulfite amplicon sequencing (BA-seq). The heatmaps exemplify the presence of methylated (red) and non-methylated (blue) CpGs within the *PDE4C* amplicon, covering 26 neighboring CpGs. The age-associated CpG of the aging signature is indicated by arrow. Exemplary heatmaps are depicted for e) a healthy donor, and f) a PMF patient blood sample of the same age. The frequency of reads is clustered according to their DNAm patterns. The same analysis was performed in colony-forming units (CFUs) on day 14 that were either g) a wild type (WT), or h) harbored the *JAK2* V617F mutation. Unlike the blood samples from PMF patients or controls, the CFUs exhibited a distinct DNAm pattern that appears to reflect the clonal characteristics of the colony-initiating cells

Subsequently, we investigated if the acceleration of epigenetic age predictions in PMF varied among samples with different driver mutations. Building on our earlier research using bisulfite amplicon sequencing (BA-seq) of three age-associated regions of *PDE4C, FHL2*, and *CCDC102B*, which indicated an overall acceleration of epigenetic age in MPN [24], we employed two epigenetic signatures [25, 26] to further validate that PMF exhibits significantly accelerated epigenetic age, consistent across all three driver mutations (Fig. 4a–d). The difference between predicted and chronological age (delta-age) did not correlate with mutation burden.

To delve deeper into the heterogeneity of epigenetic aging, we revisited the BA-seq data of amplicons within the three age-associated regions. Notably, the DNAm at neighbouring CpGs within individual reads of amplicons appeared to be independently regulated (Fig. 4e, f; Supplemental Figure S4a), corroborating previous findings in healthy samples [25]. However, in a clonal disease context, we expected to see a dominant pattern reflective of the tumor-initiating cells. Consequently, we examined BA-seq DNAm patterns at the same age-associated CpGs in single-cell-derived colony-forming units (CFUs; Fig. 4g, h; Supplemental Figure S4b). In fact, we observed prominent patterns in CFUs, regardless of whether they are derived from cells with or without *JAK2* V617F mutation, indicating that age-associated DNAm patterns remain largely preserved at least during the CFU formation.

### Disease-associated DNAm and cellular composition

**Fig. 5:**
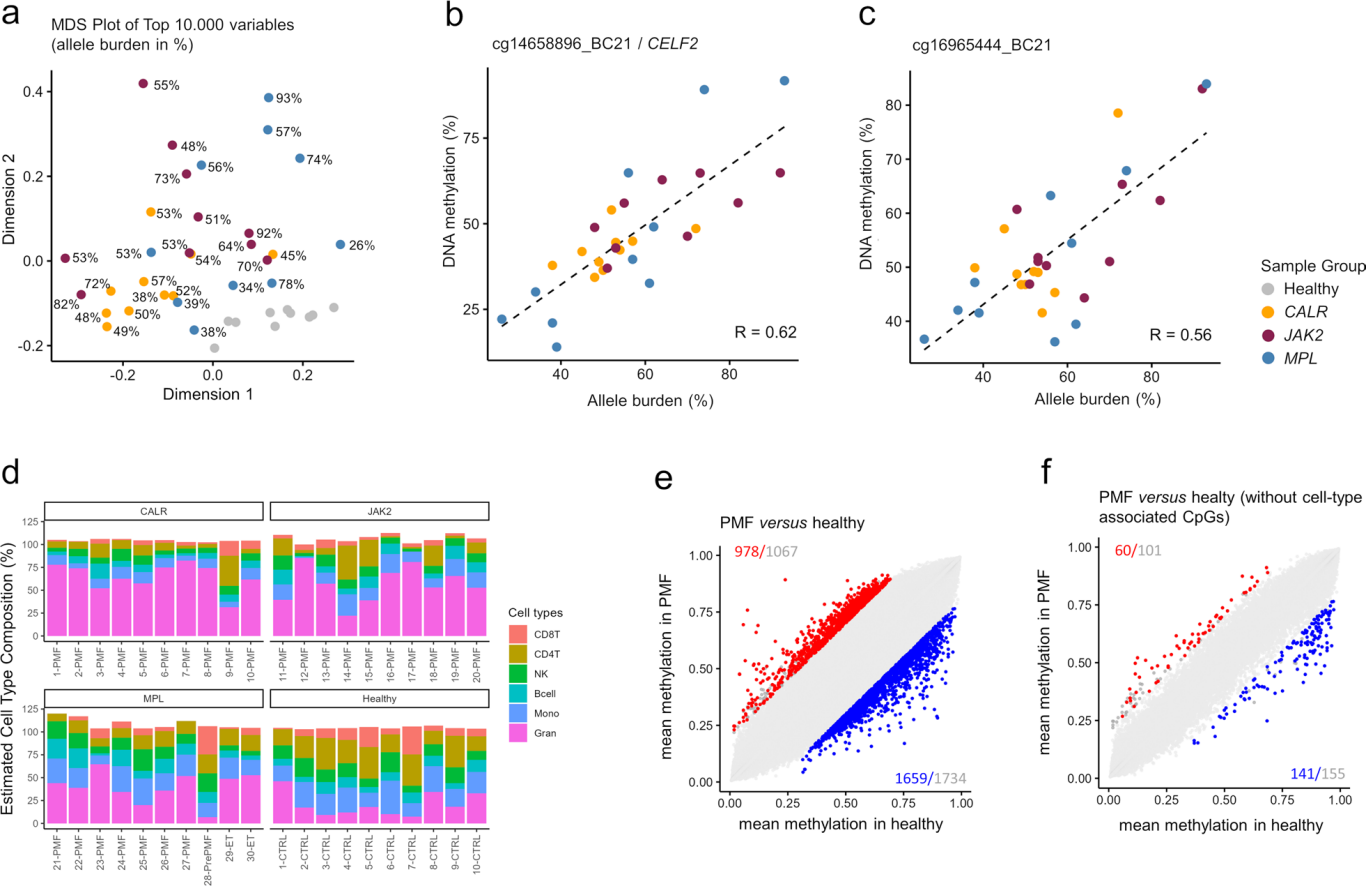
DNA methylation changes are largely attributed to the cellular composition. **a** The multidimensional scaling plot demonstrates that PMF sample did not cluster by mutation allele frequency. **b**, **c** Correlation between DNAm and allele burden (across all driver mutations, given that they hardly affected DNAm) for the top two candidate CpGs: **b**) cg14658896_BC21 and c) cg16965444_BC21. **d** An epigenetic deconvolution algorithm [51] was applied to estimated fractions of granulocytes, monocytes, B cells, NK cells, CD4 and CD8 T cells. **e**, **f** To determine how many significant DNAm changes in PMF *versus* controls is attributed to CpGs that have high variation between leukocyte subsets we compared scatter plots e) before and f) after exclusion of CpGs with more than 10% DNAm between any of the leukocyte subsets (only CpGs measured across all datasets are shown). Significant hypo- and hypermethylated CpGs are indicated in blue and red (mean DNAm difference > 20%; adjusted *p*-values < 0.05). Gray numbers indicate all CpGs exceeding the mean DNAm difference > 20%, irrespective of statistical significance

We hypothesized that the allele burden of driver mutations serves as a proxy for the fraction of malignant cells, potentially correlating with PMF-associated aberrant DNAm. In blood samples from PMF patients, allele frequencies ranged from 26 to 93%, although these values were likely different in the PBMCs due to depletion of granulocytes. Multidimensional scaling analysis revealed minimal clustering of DNAm profiles according to allele burden (Fig. 5a), suggesting that aberrant DNAm may not be homogeneous across malignant clones. Furthermore, only few individual CpGs exhibited moderate correlation with the allele burden of *JAK2* V617F, *CALR*, or *MPL* mutations (Supplemental Table S5). For example, cg14658896_BC21 (*R* = 0.62) and cg16965444_BC21 (*R* = 0.56) displayed an association with allele burden, irrespective of the specific driver mutation (Fig. 5b, c).

So far, it is largely unclear how much of the aberrant DNAm in myeloid malignancies can be attributed to the changes in the cellular composition of blood. While precise flow cytometric analysis or manual cell differential counts were not available for our samples, we used a deconvolution algorithm for cell type-specific DNAm signatures [27]. This analysis revealed higher predictions for granulocytes in PMF samples, as compared to controls, albeit PBMCs were used for both groups (Fig. 5d). To further explore this granulocyte bias, we alternatively applied the deconvolution algorithm to triple negative MPN samples (GSE156546) [28] and subsets of hematopoietic stem and progenitor cells (GSE63409) [11]. These results suggested that myeloid bias of predictions in MPN and the increased fraction of progenitor cells might contribute to higher estimates of granulocytes in the deconvolution algorithm (Supplemental Figure S5).

Given the significant differences in cellular composition between PMF patients and healthy donors, we subsequently concentrated on CpGs exhibiting stable DNAm levels across all healthy leukocyte subsets. This approach was taken to minimize the impact of disparate cellular compositions. Analyzing 289 DNAm profiles from sorted cell types (Supplemental Table S2), we excluded CpGs with over 10% DNAm variation in pairwise comparisons. This process yielded 393,675 CpGs with consistent DNAm across various healthy donor cell types. Comparing PMF and control samples, we found 978 CpGs significantly hypermethylated and 1,659 hypomethylated in PMF (adj *p* < 0.05; mean DNAm difference > 20%; Fig. 5e). However, after excluding CpGs with high-variation between leukocyte subsets, only 60 were significantly hypermethylated and 141 hypomethylated (Fig. 5f), indicating that while many PMF-associated epigenetic aberrations are linked to cellular composition, some remain distinctive.

### Comparative epigenetic analysis with other myeloid malignancies

**Fig. 6:**
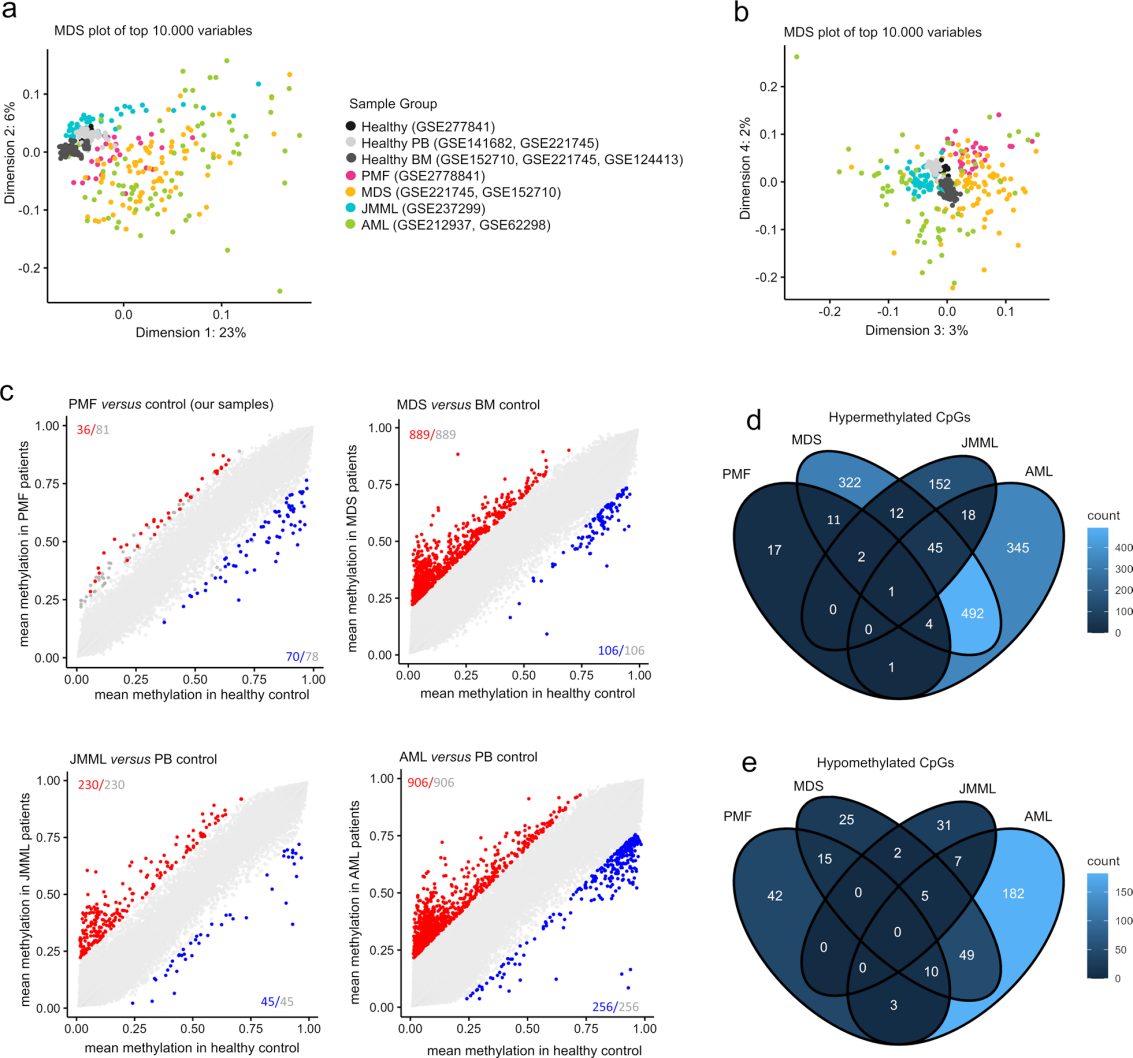
Comparison of myeloid malignancies after exclusion of cell type-specific CpGs. **a**, **b** Multidimensional scaling plots of DNAm profiles (216,532 CpG sites) in patients with PMF, MDS, JMML, AML, and healthy controls (peripheral blood (PB) and bone marrow (BM)): a) first *versus* second dimension; b) third *versus* fourth dimension. **c** Scatter plots showing mean DNAm beta values of healthy control *versus* PMF, MDS, JMML, or AML, accounting for potential differences between peripheral blood and bone marrow by using appropriate control sets. Significant hypo- and hypermethylated CpGs are indicated in blue and red (mean DNAm difference > 20%; adjusted *p*-values < 0.05). Gray numbers indicate all CpGs exceeding the mean DNAm difference > 20%, irrespective of statistical significance. **d**, **e** Venn diagrams illustrating CpGs that are overlapping d) hyper- or e) hypomethylated in the above-mentioned comparisons of four myeloid malignancies after the exclusion of cell type-specific CpGs

To examine how DNAm profiles in PMF patients compare with other myeloid malignancies, we analyzed datasets from bone marrow samples of myelodysplastic syndrome (MDS) patients [29, 30], peripheral blood of juvenile myelomonocytic leukemia (JMML), and peripheral blood from acute myeloid leukemia (AML) patients [31, 32]. To account for potential biases related to tissue type, we compared these against corresponding healthy controls (Supplemental Table S3). Multidimensional scaling showed that healthy samples clustered closely together, while JMML formed a distinct cluster. PMF, MDS, and AML profiles did not separate clearly; however, PMF samples tended to cluster closer to healthy samples, followed by MDS and AML (Fig. 6a,b). A similar pattern was observed when we excluded the CpGs with high variability between leukocyte subsets (Supplemental Fig. 6a, b).

Subsequently, we analyzed pairwise comparisons of DNAm changes between individual diseases and compounding controls (either BM or PB). Without filtering for cell type-specific CpGs, we identified many significant CpGs for each disease (adj *p* < 0.05; difference of mean DNAm > 20%; Supplemental Fig. 6c), and there was a considerable overlap in CpGs that were hyper- or hypomethylated across PMF, MDS, JMML, and AML (Supplemental Fig. 6d, e). However, when we excluded CpGs with high variability between cell types, the differential DNAm signatures for each disease became significantly reduced and overlap among myeloid malignancies was minimal (Fig. 6c–e). Only one CpG site (cg04470072_TC11) was hypermethylated across all these malignancies. Thus, while the DNAm changes in comparison with healthy blood samples can be largely attributed to the cellular composition, certain epigenetic aberrations may be indicative of specific diseases.

### Establishing an epigenetic signature for PMF

To determine if PMF possesses unique DNAm patterns that could aid in diagnosis, we performed pairwise comparisons between PMF and other myeloid malignancies, identifying significant differences in 387 CpGs for MDS, 700 for JMML, and 689 for AML (Supplemental Fig. 7a–c). Among these, 17 CpGs were overlapping hypermethylated and 36 hypomethylated which are specific for PMF (Supplemental Fig. 7d, e). Next, we analyzed if these CpGs were also differentially methylated in PMF *versus* heathy controls (Fig. 6d, e). In fact, six hypomethylated CpGs (none of the hypermethylated CpGs) were overlapping in these comparisons: cg02210934 (no gene), cg02739280 (*NAV2*), cg21708058 (*TACC1*), cg07197092 (no gene), cg08069247 (*HABP2*), and cg04902833 (*C17orf99;* Supplemental Fig. 8a). However, none of these CpGs could reliably discern all PMF from other samples. Furthermore, cg04902833 (*C17orf99*) was excluded from further analysis, because it was also hypomethylated in our control samples, indicating that it might be affected by batch effects or microarray versions.

**Fig. 7:**
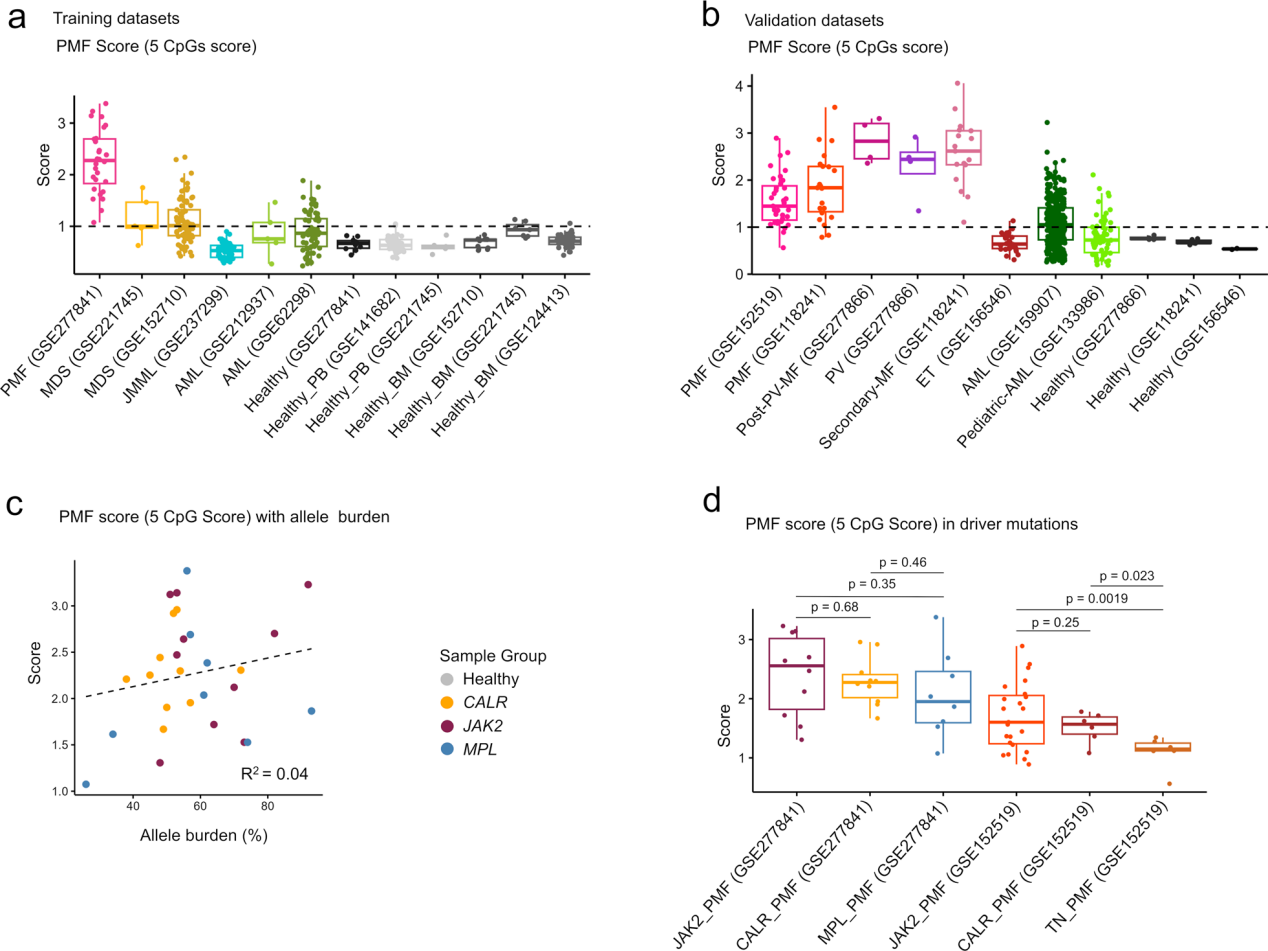
A five-CpG signature can discern PMF from other malignancies and controls. **a**, **b** The DNAm levels at five CpGs (cg02210934, cg02739280, cg21708058, cg07197092, and cg08069247) were combined into a simple PMF score (PMF score = 5—sum of the five DNAm values). The PMF score is provided for datasets of the a) training set, and b) independent validation set. The datasets of PMF, other myeloid malignancies and healthy samples used from both our and public datasets are indicated with GSE numbers. **c** The PMF score did not correlate with mutation allele burden. **d** Box plot demonstrating that the driver mutations did not have significant impact on the PMF score. However, triple negative (TN) samples of publicly available PMF datasets (GSE152519) revealed a lower PMF score. Statistical significance was determined using an unpaired *t*-test

Since single CpG analysis was insufficient for effective differentiation, we combined the remaining five CpGs into a PMF score (calculated as 5 minus the sum of the beta values, with higher scores indicating a stronger association with PMF). This score was not intended for clinical application, but rather to test if DNAm patterns can be used to discern MPN from other myeloid malignancies. In fact, the 5 CpG signature could effectively distinguished PMF samples from all controls and most myeloid malignancies (Fig. 7a). To further validate the score, we utilized other available datasets of PMF [7, 17], secondary myelofibrosis [17], ET [28], AML [33], pediatric AML [34], additional healthy controls, and own yet unpublished 450 k profiles of post PV-MF, PV, and healthy controls (Supplemental Table S3). All five CpGs of the PMF score showed clear offsets in MPN samples, except for the ET samples (Supplemental Fig. 8b). The PMF score was higher (> 1) in almost all PMF, MF and PV samples (Fig. 7b). Importantly, the PMF score did not correlate with the allele burden of driver mutations, reinforcing our earlier finding that epigenetic aberrations are heterogeneous within the malignant clone (Fig. 7c). Furthermore, PMF score did not reveal significant differences in our samples with *JAK2* V617F, *CALR*, and *MPL* mutation. Notably, in comparison with a public dataset of triple negative (TN) PMF samples, the PMF score was lower in TN than in *JAK2* V617F or *CALR* mutated samples (Fig. 7d).

## Discussion

The results of this study indicate that the driver mutations in PMF have surprisingly little impact on the disease-associated epigenetic modifications. Significant DNAm differences between PMF with *JAK2* and *CALR* mutations were scarce, consistent with a recent study in ET patients [28]. In contrast, the observed differences in *MPL* mutations might partly be attribute to lower allele burden, inclusion of two ET samples, and inclusion of one sample with a low additional allele frequency of an additional *CALR* mutation in this group. Moreover, the *MPL* comprised nine of ten male samples—since there are also moderate gender-related DNAm differences on autosomes this might also contribute to the discrepancy to the *JAK2* and *CALR* groups [35, 36]. The molecular link between the genomic and epigenetic modifications remains unclear. All three driver mutations activate the JAK-STAT signaling pathway, and while *JAK2* and *CALR* mutations were suggested to cause distinct mitotic defects leading to chromosomal instability [37], *MPL* mutation directly affect the structure and function of thrombopoietin receptor [38, 39].

When we investigated the impact of the *JAK2* V617F mutation in iPSCs, we observed no significant epigenetic changes. The moderate association of *JAK2* V617F PMF-associated DNAm changes in iHPCs might also be attributed to the pathognomonically skewed megakaryocytic and erythroid differentiation of iPSCs with the *JAK2* V617F mutation [21, 22]. It is conceivable that time of hematopoietic differentiation in vitro does not suffice to evoke mutation-associated DNAm changes—particularly given that MPN seems to usually develop over decades. Thus, the iPSC model may not be ideally suited to recapitulate the complex epigenetic modifications that arise over many years during development of the disease—at least, it did not reflect an immediate sequel of MPN-associated mutations on corresponding DNAm changes.

Additionally, the PMF-associated DNAm changes did not correlate with mutational allele burden, indicating that the epigenetic modifications are not homogenous in the entire malignant clone. Only very few CpGs correlated with allele burden, and it might be speculated that these arise earlier after the driver mutation and hence better reflect fraction of the malignant clone. Furthermore, the epigenetic makeup can also be altered in the non-malignant hematopoietic compartments or in the bone marrow microenvironment [40].

Previous studies suggested that alterations in the DNAm landscape play an important role in the pathogenesis and leukemic transformation of MPN [6]. This might be caused by additional mutations in epigenetic writers, such as *ASXL1, DNMT3A*, *SRSF2*, and *TET2* [39, 41–43]. Either way, the PMF-associated epigenetic changes were observed across different secondary mutations and many of these mutations are also frequently observed in other malignancies. This suggests that secondary mutations in epigenetic writers are not the sole drivers of the epigenetic changes seen in PMF.

Given that different cell types exhibit distinct DNAm profiles, it is crucial to consider cellular heterogeneity when interpreting DNAm data in hematological diseases [44–46]. We excluded CpGs that displayed variability among different leukocyte subsets, demonstrating that previously noted differences in comparisons of diseased *versus* healthy blood largely stem from cellular composition. Furthermore, epigenetic aberrations have hardly been compared across different myeloid malignancies [47–49]. Our integrative analysis across multiple studies demonstrated that also such comparisons are largely affected by differences in the cellular composition.

We subsequently followed the question if PMF-associated DNAm patterns are disease specific and how they are related to other myeloid malignancies. None of the individual CpGs could reliably discern PMF from healthy controls as well as from other myeloid malignancies, underscoring the need to combine multiple CpGs into comprehensive epigenetic signatures. Furthermore, there was a gradual overlap of aberrant DNAm in PMF with all other hematopoietic malignancies. Our pairwise comparisons indicate that there are significance differences in the methylome of PMF, MDS, AML, and JMML samples. However, such analysis can be biased by many parameters, including differences in the cellular composition, allele burden, gender, array types and batch variation between different studies. Thus, further analysis is needed to elaborate the characteristic epigenetic phenotype of hematopoietic malignancies.

As a proof of concept, we have exemplarily generated a 5 CpG PMF score to discern PMF from other hematopoietic malignancies. However, it could not discern PMF and PV samples. Either way, our score has not been validated and optimized for clinical application. Looking ahead, such scores could ultimately be used to support stratification of specific MPN entities or diagnosis of triple negative cases. Further refinements may even reveal the transition of MPN into a secondary AML. Small epigenetic signatures may facilitate a clinical translation, because they enable targeted analysis, using methods such as pyrosequencing, BA-seq, or digital PCR, to facilitate fast and cost-effective assessments for clinical application [50]. However, given the heterogeneity of aberrant methylation patterns that seem to evolve rather independent from driver mutations, it appears to be necessary to consider larger signatures to reliably discern hematopoietic diseases.

## Conclusions

The results of this study demonstrate that epigenetic patterns can differentiate myeloid malignancies, with the observed differences not directly linked to specific driver mutations but rather influenced by cellular composition and overlapping across various myeloid diseases.

## Limitations

Our study is limited in that we did not analyze the methylome of sorted and defined hematopoietic subpopulations—ideally sorted as healthy and malignant cell fractions directly from the bone marrow. In fact, our results demonstrate that many epigenetic aberrations in myeloid malignancies can be attributed to cell type-specific DNA methylation changes. The use of PBMCs, absence of conventional leukocyte counts, and the relatively small samples sizes are further limitations. While PMF with *JAK2* and *CALR* mutation have similar epigenetic aberrations, it needs to be further explored why our *MPL* samples had less pronounced aberrations. At this point, we could not identify reliable epigenetic patterns for specific driver mutations or MPN entities that could be applied for clinical application.

## Supplementary Information

Supplementary file 1. Figures S1-S8, Table S1 and S6.

Supplementary file 2. Table S2. 

Supplementary file 3. Table S3.

Supplementary file 4. Table S4.

Supplementary file 5. Table S5.

## Data Availability

The DNAm data on PMF (EPIC v2) and MPN (450 k) are accessible in the Gene Expression Omnibus (GEO) under super series GSE277889 with sub series GSE277841, and GSE277866. Additionally, DNAm profiles for JAK2 iPSCs and iHPCs (EPIC v1) can be found under GSE277890.
